# Integrated Biomass Valorization: From Platform Molecules to Functional Carbon Materials for Sustainable Energy and Environmental Applications

**DOI:** 10.1021/acsomega.6c03202

**Published:** 2026-06-22

**Authors:** Armanda A. Júlio, Amanda C. Filgueiras, Julianne A. Bruno, Garbas A. dos Santos Junior, Patrícia F. Pinheiro

**Affiliations:** Department of Chemistry, Universidade Federal de Viçosa, Avenida Peter Henry Rolfs, s/n, 36570-900 Viçosa, Minas Gerais, Brazil

## Abstract

The increasing demand for sustainable alternatives to fossil-based resources has intensified interest in the integrated valorization of lignocellulosic biomass within the context of biorefinery and circular economy strategies. However, the relationship between biomass-derived platform molecules and biomass-derived carbonaceous materials remains insufficiently explored in the literature. This review discusses recent advances in the conversion of biomass into platform molecules, particularly furfural and 5-hydroxymethylfurfural (HMF), with emphasis on catalytic pathways and the role of homogeneous, heterogeneous, and biomass-derived catalysts in controlling process selectivity and efficiency. Representative studies reporting HMF yields above 90% and recyclable biomass-derived catalysts are highlighted to demonstrate the potential of sustainable catalytic approaches. In parallel, the synthesis of biomass-derived carbonaceous materials via pyrolysis and hydrothermal carbonization is discussed, together with surface functionalization strategies involving sulfonic, amino, and phosphate groups. These modifications enable the development of multifunctional materials for applications in catalysis, electrochemical systems, and adsorption processes. Furthermore, the review highlights the integrated role of biomass as both feedstock and catalyst precursor, reinforcing the concept of closed-loop biomass valorization. Finally, current challenges related to catalyst stability, process scalability, and sustainable industrial implementation are critically discussed.

## Graphical abstract

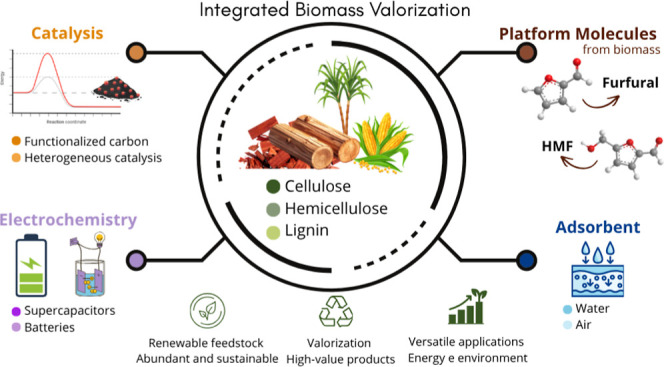

## 1 Introduction

Global demand for energy and raw materials has increased significantly, intensifying the consumption of fossil resources and contributing to greenhouse gas emissions and environmental impacts.^1^ This scenario underscores the urgency of transitioning toward sustainable energy systems and greener chemical processes based on renewable resources.^2,3^

Biomass has emerged as a promising alternative due to its abundance, renewability, and high carbon content, with the potential to replace fossil-based feedstocks in multiple industrial applications.^1,4^ In particular, lignocellulosic biomass is attractive because of its wide availability and its noncompetition with food production, as it is mainly derived from agricultural, forestry, and industrial residues.^5^

Building on this potential, biomass can be converted into strategic platform molecules such as furfural, 5-hydroxymethylfurfural (HMF), levulinic acid, and polyols, which serve as versatile intermediates for fuels, fuel additives, and sustainable polymers.^1,6^ This approach aligns with the biorefinery concept, in which biomass fractions are transformed into value-added products through chemical, biological, or thermochemical routes.^7^

Complementarily, biomass has also been widely explored as a precursor for functional carbonaceous materials, establishing a direct link between molecular valorization and materials design. Thermochemical processes such as pyrolysis and hydrothermal carbonization yield biochar and hydrochar, respectively, with tunable porosity and surface area.^8,9^ Subsequent chemical activation further tailors their acid–base, electronic, and structural characteristics.^10,11^

These biomass-derived materials have shown strong performance in catalytic processes for fuel production,^12^ hydrogen generation,^13^ and electrochemical energy storage.^14^ In addition, recent studies have demonstrated that such materials can be rationally engineered into advanced functional systems, including donor–acceptor conjugated architectures for photocatalytic applications.^15,16^ These systems enable enhanced charge separation and tunable electronic properties, expanding their applicability in solar-to-chemical energy conversion. Their low cost, renewability, scalability, and tunable structural properties position them as key enablers in the transition toward sustainable energy systems.

Thus, this review goes beyond conventional approaches by integrating two strands often addressed separately: the production of energy-relevant platform molecules and the design of biomass-derived functional carbon materials. By explicitly linking molecular-level biomass conversion pathways to the development of advanced carbon systems, it provides a unified framework for biomass valorization.

This perspective is further reinforced by the discussion of practical applications, in which biomass-derived carbon materials are explored as catalysts, catalyst supports, and components in electrochemical systems and adsorption processes.

This integrated framework consolidates recent advances and identifies key challenges and design principles for next-generation materials, positioning biomass as a central pillar of scalable, low-carbon technologies aligned with green chemistry and the circular economy.

## 2 Biomass: Source, Structure, and Potential as Chemical Platform

Biomass is a valuable and promising renewable resource for producing fuels, chemicals, and sustainable materials. A variety of sources can be utilized, and they can be classified into categories such as agricultural residue (e.g., straw, husks, pruning waste, fruits peels),^17^ forestry biomass (e.g., wood, bark, sawdust),^18^ and byproducts from the timber and pulp and paper industries (e.g., lignin-rich residues).^19^

The source of biomass plays a crucial role in determining its chemical composition and structural organization, which directly influence its suitability for catalytic and energy applications. Among these, lignocellulosic biomass stands out as the most abundant class, being primarily composed of cellulose (30–50 wt %), hemicellulose (20–40 wt %), and lignin (15–25 wt %)^20^ (Figure 1).

**Figure 1:**
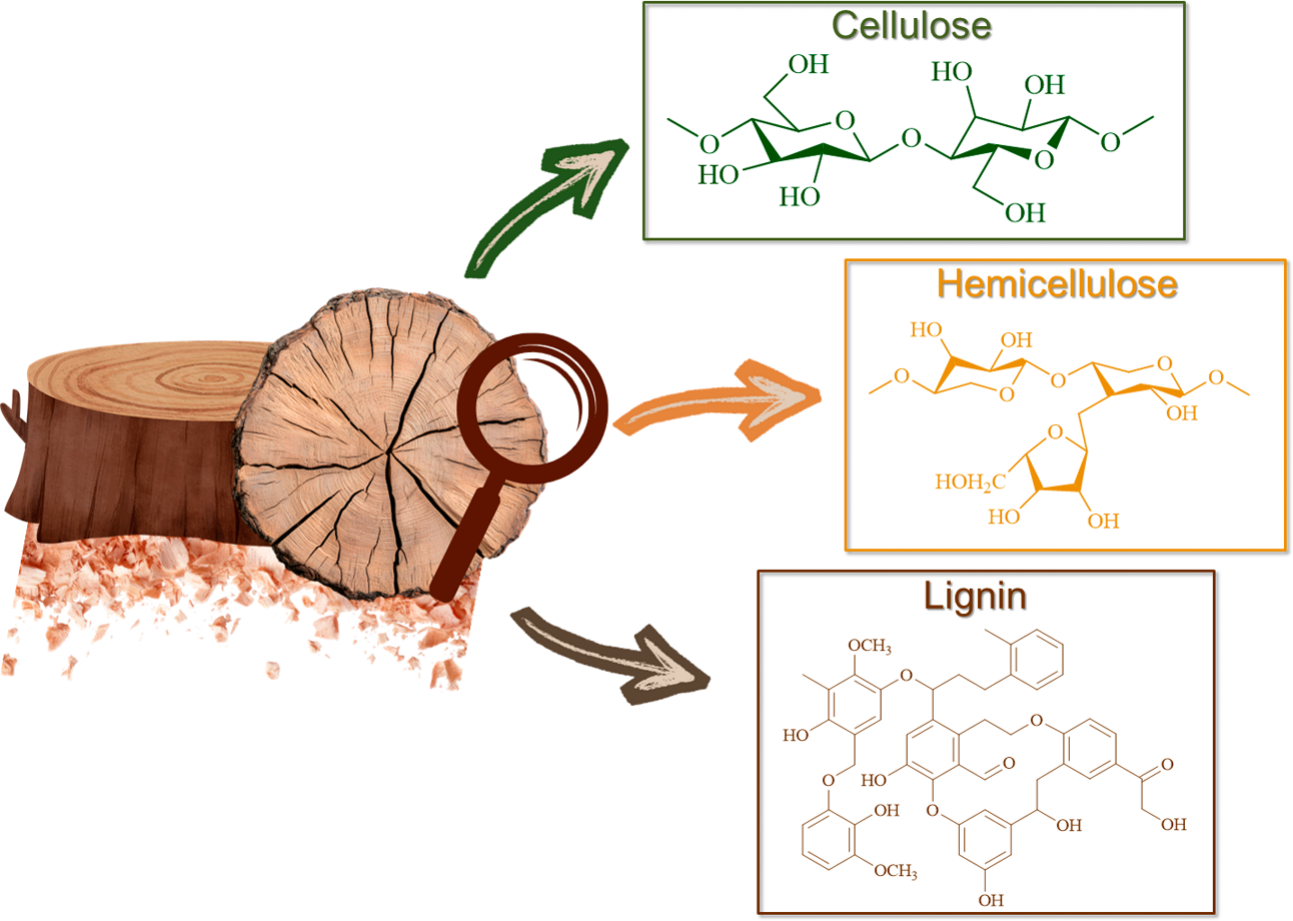
Main structural components of lignocellulosic biomass.

However, the relative proportions of these components vary significantly depending on the biomass source. Agricultural residues are typically richer in cellulose and hemicellulose, making them more amenable to conversion into chemical platforms.^21,22^ In contrast, forestry biomass generally exhibits a higher lignin content, favoring the production of aromatic-rich carbon materials and functionalized biochars.^22^

This compositional variability plays a decisive role in determining the physicochemical properties of derived materials, such as porosity, surface functionality, and thermal stability, which directly impact their performance in catalytic applications. Therefore, a clear classification and comparison of biomass sources are essential to rationally guide their valorization into functional carbon materials.

At the molecular level, cellulose is a linear polymer of glucose containing both crystalline and amorphous regions and represents the fraction most resistant to degradation. Its microfibrils are closely associated with hemicellulose, an amorphous polysaccharide composed mainly of pentoses and hexoses, which are more susceptible to hydrolysis. These polysaccharide fractions constitute the main precursors of sugars that, after catalytic dehydration, can be converted into high-value platform molecules such as furfural and HMF.^23,24^

Lignin, which surrounds the hemicellulose-cellulose matrix, forms a three-dimensional aromatic network rich in C–C and C–O linkages and is largely responsible for the recalcitrance of lignocellulosic biomass.^23,24^ This high aromatic carbon content also makes lignin and thermochemical residue suitable precursors for carbonaceous materials.

### 2.1 Integrated Biomass Valorization: Platform Molecules and Carbonaceous Materials

Among the strategies applied for the transformation of polysaccharides derived from lignocellulosic biomass, the production of furfural and HMF represents one of the most important pathways, as these molecules serve as key building blocks for the synthesis of a wide range of chemicals. Figure 2 illustrates the formation of these two compounds from the hydrolysis of hemicellulose and cellulose.

**Figure 2:**
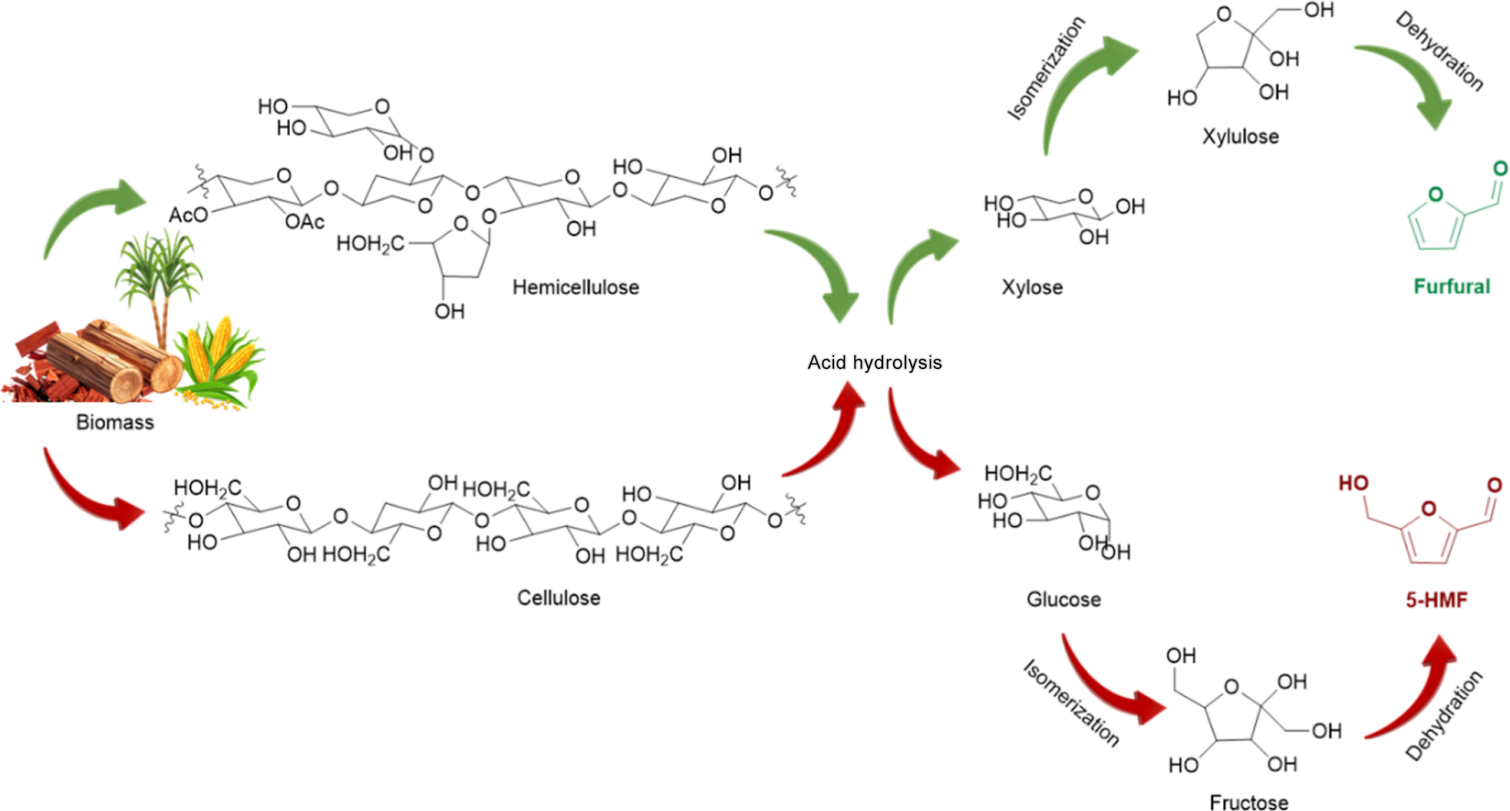
Conversion pathways for the formation of furfural and 5-hydroxymethylfurfural (HMF) from lignocellulosic polysaccharides.

Biomass undergoes pretreatment to release cellulose, hemicellulose, and lignin fractions, enabling their conversion into higher-value products. Hemicellulose and cellulose serve as key sources of platform molecules such as furfural and HMF. Their depolymerization typically proceeds via acid hydrolysis. To illustrate this process, the mechanism of furfural production over acid catalysts is shown in Figure 3. Upon acid-catalized cleavage of β-1,4-glycosidic bonds in hemicellulose and cellulose, H^+^ promotes the formation of C5 pentoses (e.g., xylose and arabinose) and C6 hexoses (e.g., glucose and fructose), respectively.^25^ Inorganic acids, such as H_2_SO_4_, are commonly employed as catalyst in the hydrolysis and dehydration steps involved in these transformations.^26,27^

**Figure 3:**
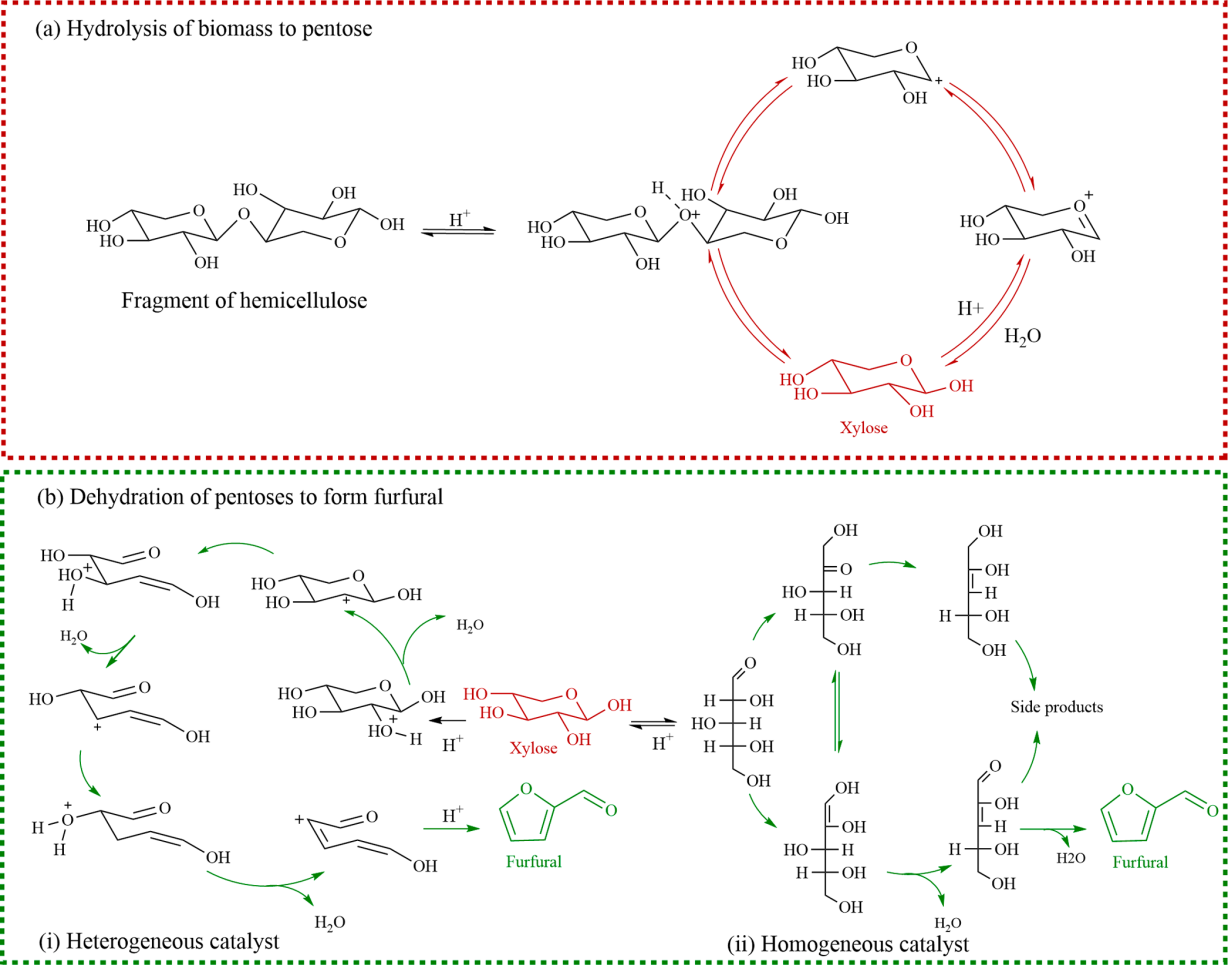
Mechanism of furfural production with acid catalysts. (a) Hydrolysis of biomass to pentose (b) dehydration of pentose to furfural with (i) heterogeneous catalysis and (ii) heterogeneous catalysis. Source: adapted with permission from ref 25 Licensed under a Creative Commons Attribution 4.0 International License, CC BY.

Following biomass hydrolysis, pentoses undergo acid-catalyzed dehydration to form furfural. In homogeneous systems, pentose is initially isomerized into enediol intermediates under the action of Lewis acids, followed by successive dehydration steps leading to cyclization, and the formation of furaldehyde in the presence of Brønsted acids. In heterogeneous systems, the mechanism involves protonation of hydroxyl groups, followed by water elimination, carbocation formation, and structural rearrangements until final cyclization occurs, yielding furfural. Thus, the type of catalyst directly influences both the reaction mechanism and its efficiency.^25^

High xylose conversion (82–96%) to furfural (28–38% yield) can also be achieved using more sustainable catalysts, such as biomass-derived carbon-supported solid acids; however, further optimization is still required to improve process selectivity.^28^ In sustainable systems, sulfonated activated carbon has promoted the dehydration of fructose with up to 100% yield of HMF, and after four reaction cycles, the yield remained as high as 94%.^29^ These studies highlights the versatility of biomass and its potential within the circular economy, as biomass-derived compounds are converted using catalysts that are themselves derived from biomass.

Other recent studies have reported several strategies to improve the yield of furfural and HMF from biomass by combining advances in catalytic systems, reaction media, and process design. Continuous-flow approaches have demonstrated particular relevance for process intensification and scalability. For instance, Megbenu et al. (2025),^30^ achieved furfural yields of approximately 74% from xylose, using a ZnCl_2_/NaCl catalytic system, although lower yields were observed for lignocellulosic residues such as corn cob and rice husk. Similarly, Ariyanti et al. (2025)^31^ reported furfural yields exceeding 93% from corn cob using AlCl_3_, highlighting the effectiveness of Lewis acid catalysis in real biomass feedstocks and its alignment with circular economy strategies.

In addition to catalytic design, the reaction medium and processing route play a critical role, as demonstrated by Rakhatkyzy et al. (2024)^32^ compared continuous-flow and microwave systems for HMF production using deep eutectic solvents, achieving significantly higher yields under continuous flow conditions. Collectively, these studies demonstrate that the interplay between catalyst selection, reaction environment, and process configuration is essential for advancing efficient and scalable biomass valorization strategies.

In addition to catalytic conversion into platform molecules, biomass can also be valorized through carbonization routes. Depending on the methodology employed, acid (e.g., –SO_3_H) or basic (e.g., –NH_2_) functional groups can be introduced onto the carbon surface, and metallic particles may be incorporated to form hybrid materials with tailored catalytic properties. Furthermore, carbonized biomass can serve as an efficient catalytic support, thereby expanding its applicability in sustainable chemical processes.

In this context, the following sections discuss strategies for the catalytic conversion of furfural and HMF, as well as the synthesis and functionalization of biomass-derived carbonaceous materials.

### 2.2 Furfural

Furfural is one of the main platform molecules derived from lignocellulosic biomass, and the demand for this feedstock has increased significantly in recent years. Its production from agricultural residues, such as bagasse and straw^33^ reinforces its role within the context of sustainable biorefineries.

Owing to the presence of reactive functional groups (a furan ring and an aldehyde group), furfural is a highly versatile molecule and can be catalytically converted into a wide range of industrially relevant products, as illustrated in Figure 4.

**Figure 4:**
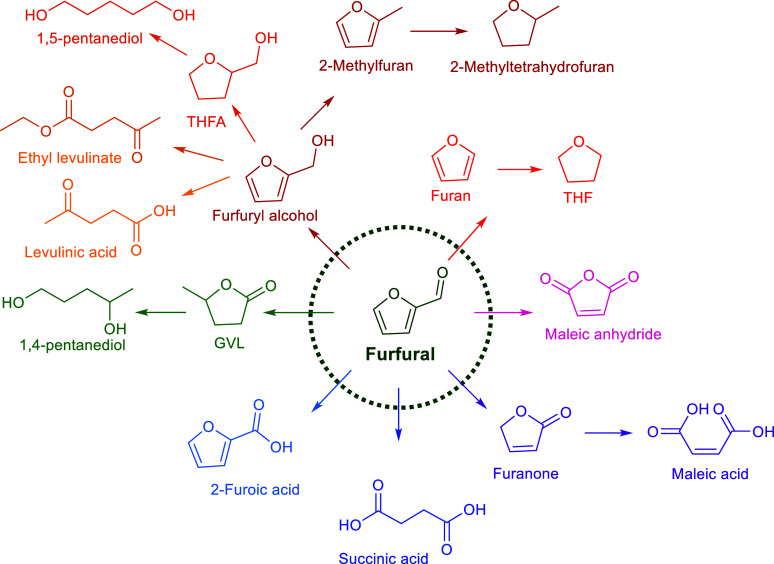
Representative products obtained from the catalytic conversion of furfural.

Furan can be obtained from the decarboxylation of furfural and subsequently hydrogenated to produce tetrahydrofuran (THF), which can be used as a solvent or as a precursor in polymer production. Studies have reported high yields (up to 88%) of furan from furfural decarboxylation when catalyzed by Ni/Mg-based solid catalysts.^34^ The subsequent hydrogenation of furan to THF is commonly catalyzed by Pd- or Pt-based catalysts.^35^

The hydrogenation of furfural leads to the formation of furfuryl alcohol, which can undergo hydrodeoxygenation to produce 2-methylfuran, a molecule that can participate in hydroxyalkylation–alkylation reactions to generate sustainable aviation fuels (SAFs).^36^ Subsequent hydrogenation of 2-methylfuran yields 2-methyltetrahydrofuran (2-MTHF), which is considered a versatile and green solvent, as well as a potential alternative to conventional petroleum-based solvents.^37^

The hydrogenation of furfuryl alcohol can also lead to the formation of tetrahydrofurfuryl alcohol (THFA),^38^ which can subsequently undergo dehydratation to produce 2-MTHF.^39^ THFA is an important intermediate in the synthesis of various polymers with industrial applications.^40^

When furfuryl alcohol undergoes alcoholysis, alkyl levulinates such as ethyl levulinate are formed, which are widely applied as biofuel additives.^12^ Levulinic acid can also be obtained from the conversion of furfuryl alcohol and serves as an important intermediate for the synthesis of levulinates.^41,42^ Furfuryl alcohol is therefore an important industrial intermediate and serves as a key starting material for the synthesis of fine chemical intermediates,^43^ sustainable materials and polymers,^44^ solvent,^45^ bioadditives, and biofuels.^46,47^

γ-Valerolactone (GVL) is an important compound in the formulation of liquid fuels, as it improves octane rating and combustion efficiency and reduces NO*x* emissions when blended with fuels.^48^ It has also been reported as promising oxygenated fuel additive compared to conventional fossil-derived compounds, owing to its favorable physicochemical properties and cleaner combustion profile. In addition, GVL serves as a precursor for fine chemical, including polymers.^49^

GVL can be synthesized from furfural in one-pot systems involving multiple catalytic steps, in which Lewis and Brønsted acid sites act synergistically. For example, catalysts based on zirconium-containing metal–organic framework (MOF), with surface areas in the range of 467–528 m^2^ g^–1^, and acidity of 0.44–0.85 mmol g^–1^, promoting high conversions of furfural (>99%) with selectivity up to 83% in GVL (8 h, 160 °C, 0.01 g catalyst).^50^ Alternatively, GVL can be produced via the hydrogenation and cyclization of ethyl levulinate (with yields up to 88% over Co-based catalysts), while subsequent ring-opening and hydrogenation yield 1,4-pentanediol with up to 98% yield.^51^

Among the various transformation pathways of furfural, the formation of furfuryl alcohol, tetrahydrofurfuryl alcohol (THFA), 1,5-pentanediol, 1,4-pentanediol, 2-methylfuran, and GVL is commonly promoted by metal-containing catalysts.^25^ On the other hand, the oxidation of furfural can generate a series of value-added compounds, such as 2-furoic acid, an important product for the pharmaceutical and optical industries. Carbon-supported Ru catalysts have achieved yields of up to 83% of furoic acid.^52^

Oxidative processes can also lead to the formation of succinic acid, 2­(5*H*)-furanone, and maleic anhydride.^53–55^ Among these products, succinic acid and its derivatives stand out due to their wide range of applications in pharmaceuticals, adhesives, solvents, polymer synthesis, and food additives.^56^

Although a variety of catalytic routes have been developed for the conversion of furfural, significant challenges remain in achieving highly selective chemical transformations. These limitations arise primarily from competing side reactions, including condensation and polymerization pathways that lead to the formation of humins. Humins are insoluble oligomeric carbon structures formed via acid-catalyzed reactions of reactive intermediates (e.g., furfural and HMF), involving C–C and C–O bond formation and polymer growth.^57–59^

In parallel, these species may evolve into carbonaceous deposits (coke), especially over strong Brønsted acid sites, causing pore blocking and active site coverage.^60^ These processes promote catalyst deactivation, decreasing activity and selectivity. Mitigation strategies include tuning catalyst acidity and porosity, as well as applying regeneration treatments such as solvent washing or thermal calcination.^61^ However, these approaches often involve trade-offs between catalytic activity, stability, and process complexity, necessitating the development of more robust catalyst designs.

### 2.3 5-Hidroxymethylfurfural

HMF is primarily obtained from the dehydration of hexoses, such as glucose and fructose, in contrast to furfural, which is produced through the dehydration of pentoses. In addition, the presence of three reactive sites in the HMF molecule (the furan ring, as well as the aldehyde and hydroxymethyl groups) enables a variety of catalytic transformations, including oxidation, hydrogenation, hydrogenolysis, etherification, and acetalization. The resulting furanic derivatives exhibit important industrial and energy-related applications and are illustrated in Figure 5.

**Figure 5:**
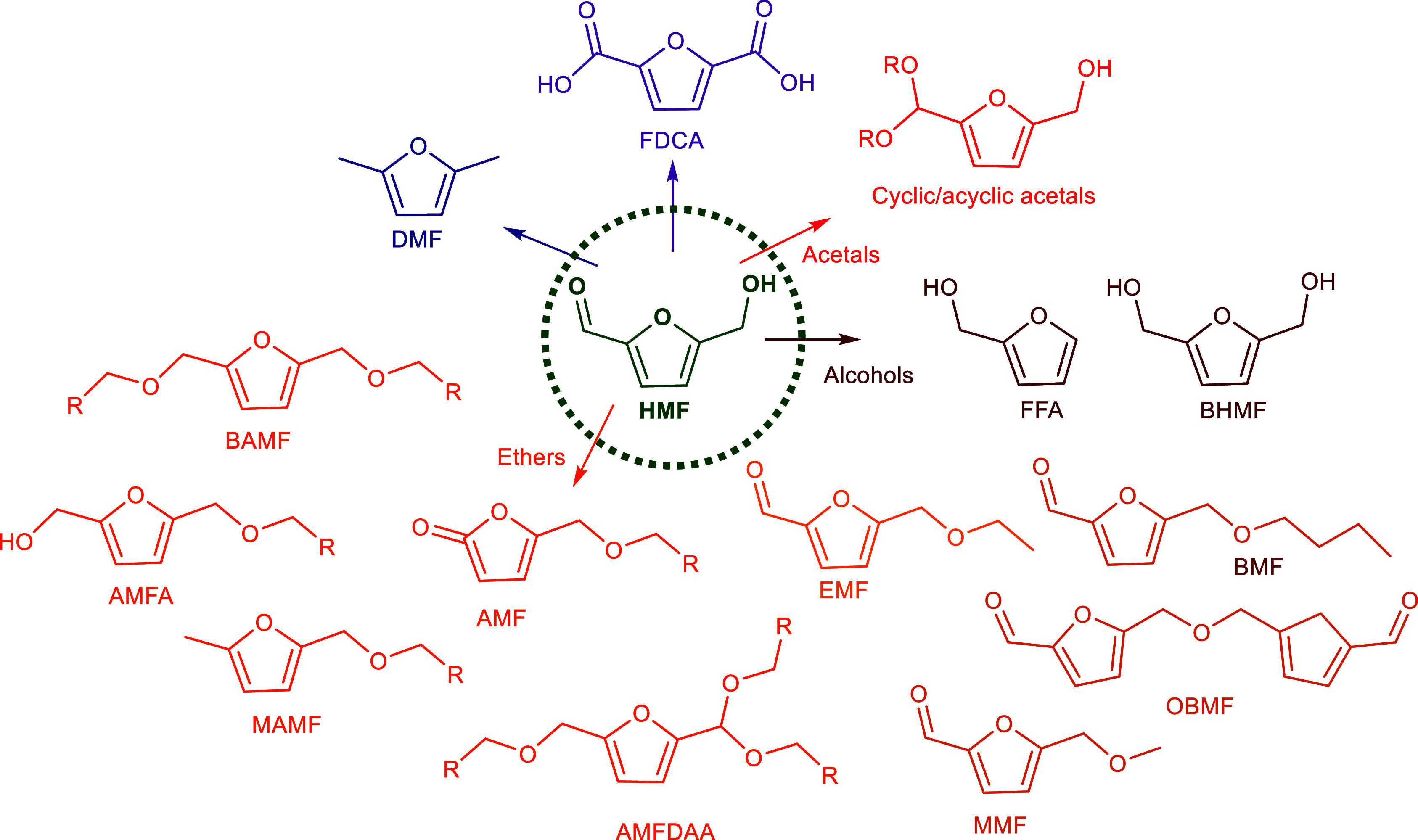
Furanic compounds derived from HMF.

HMF is considered one of the most important biobased monomers for replacing petrochemical diacids, such as terephthalic acid, in the production of renewable polyester.^62,63^ In the energy context, the hydrogenolysis of HMF to 2,5-dimethylfuran (DMF)—a liquid fuel with high energy density and properties similar to those of gasoline—makes it a promising biofuel. High yields and selectivities (>97%) have being reported using Ru–Co/activated carbon (AC) (1.5 h, 200 °C, 1 MPa),^64^ while selectivity up to 99% has been achieved using Pd-MOF (3 h, 100 °C, 1 MPa).^65^

In addition to hydrogenation routes, HMF can also be converted into furanic ethers, such as alkoxymethylfurans (AMFs) and 2,5-bis­(alkoxymethyl)­furans (BAMFs) with selectivity above 85%. These transformations typically involve sequential steps, including metal-catalyzed hydrogenation (ZnO–Fe_3_O_4_/AC, 10 h, 65 °C), followed by etherification promoted by Brønsted acidic solids.^66^ Catalysts such as protonated zeolites and Amberlyst-type resins enable high selectivities (63–95%) and conversions (∼60%) in the synthesis of ethoxymethylfurfural (EMF), a compound characterized by high energy density and improved combustion performance.^67^

In continuous-flow systems, the acid-catalyzed self-etherification of HMF (conversion up to 90%) enables the formation of products such as 5,5′-[oxybis­(methylene)]­bis-2-furfural (OBMF, selectivity 84%), along with byproducts such as 5-(methoxymethyl)­furfural (MMF, selectivity 16%), under the defined reaction conditions (60 °C, flow rate 0.3 mL min^–1^, residence time 17 min).^68^ Additionally, heteropolyacids have proven effective in promoting these transformations (89% of HMF conversion and 73% selectivity toward 5BMF), allowing structural modulation of the resulting ethers through variation of the alcohol chain employed.^69^

Other relevant routes include the selective hydrogenation of the aldehyde group of HMF, leading to the formation of 2,5-bis­(hydroxymethyl)­furan (BHMF) and related intermediates, which are important precursors for renewable resins and polymers.^70^ In addition, the formation of HMF acetals has attracted attention, as these compounds can serve as oxygenated fuel additives capable of improving combustion efficiency and reducing pollutant emissions.^71^

From a mechanistic perspective, selectivity toward desired products strongly depends on the catalytic system employed. In bifunctional catalytic systems for the conversion of biomass-derived oxygenates, metallic sites are typically responsible for hydrogen activation and hydrogenation steps, whereas acidic sites activate C–O bonds and promote transformations required for selective hydrodeoxygenation.^72^ Due to the presence of multiple reactive sites, HMF often exhibits limited selectivity toward a single product.^73^

Therefore, improving catalytic processes for the selective transformation of HMF, as well as furfural, remains one of the major challenges in biomass valorization.

### 2.4 Functionalization of Carbonaceous Materials

Various modifications can be applied to biomass-derived carbonaceous materials to tailor their structural, chemical, and surface properties, thereby expanding their applicability in catalysis, adsorption processes, and energy-related applications.^14,41,74^ Among the main modification strategies, sulfonation, amination, phosphorylation, and the formation of composite materials stand out. However, despite their functional advantages, these approaches present inherent limitations related to stability and scalability that must be critically evaluated.

Among the different functionalization approaches, sulfonation has been particularly widely explored due to its ability to introduce strong Brønsted acidity through the incorporation of –SO_3_H groups.^11^ This treatment results in materials widely applied in acid-catalyzed reactions, such as alcoholysis and acetalization, which are important processes for the production of bioadditives. For instance, sulfonated biochar derived from coffee residues has shown high yields in the formation of ethyl levulinate, while maintaining catalytic activity for up to seven reuse cycles without significant loss of performance.^75^ Similarly, sulfonated catalysts derived from date seeds have promoted high conversion of free fatty acids into biodiesel.^75^ Despite these advantages, sulfonated materials often exhibit limited stability and potential leaching of sulfonic groups,^76^ especially under aqueous or high-temperature conditions, which can lead to catalyst deactivation. Furthermore, the use of concentrated sulfuric acid, one of the most widely used sulfonating agents, raises concerns regarding process safety, corrosivity, and environmental burden, particularly at larger scales.

In addition to their catalytic applications, sulfonated carbons also exhibit high performance as adsorbents. For example, they display a notable capacity for removing potentially toxic metal pollutants, such as Pb­(II) and Cd­(II), as well as organic dyes like methylene blue.^77,78^ Similarly, sulfonated biochar is more effective for ammonium removal than its nonsulfonated counterpart.^79^ However, their performance can be influenced by solution pH, ionic strength, and temperature, as well as competitive adsorption effects, which may limit applicability in complex real wastewater matrices.^80^

Another relevant functionalization strategy is the amination of carbonaceous materials, particularly for adsorption purposes. The incorporation of nitrogen-containing groups enhances interactions with anionic pollutants through electrostatic attraction and complexation mechanisms. For example, amine-functionalized biochar obtained from watermelon rind exhibited high efficiency (98%) in the removal of Cr­(VI) from aqueous systems.^81^ Likewise, biochar derived from *Pergularia tomentosa,* modified with 5% poly­(diallyldimethylammonium chloride), showed high adsorption capacities for Acid Blue 25 (248 mg g^–1^) and Acid Red 18 (202 mg g^–1^). This performance is attributed not only to the presence of nitrogen functionalities but also to combined mechanisms such as pore filling, electrostatic interactions, hydrogen bonding, and surface adsorption.^74^

Phosphorylation has also been explored as a method for the surface modification of carbonaceous materials, potentially conferring both adsorptive and catalytic properties. While some studies report enhanced performance, such as improved U­(VI) removal using phosphate-functionalized biochar,^82^ others indicate that modification does not always result in superior adsorption capacity compared to the pristine material.^83^ This suggests that the effectiveness of phosphorylation dependent on surface chemistry, precursor selection, and functional group accessibility. Moreover, phosphate leaching under certain conditions may pose environmental risks, particularly in aquatic systems where phosphorus release can contribute to eutrophication.

In addition to direct chemical modifications, the formation of composite materials represents an important strategy for enhancing the functionality of biochar. One example is a biochar-based composite containing cobalt phosphide, whose synthesis is relatively simple and which exhibits good performance in the degradation of sulfonamide antibiotics.^84^ Composite materials often benefit from synergistic effects, including improved electron transfer, higher dispersion of active sites, and enhanced structural stability.^85^

These composites also find applications in agricultural systems. For example, the combination of biochar with carboxymethyl cellulose can form a coating for phosphate fertilizers, enabling controlled phosphorus release in soil and thereby improving nutrient uptake efficiency by plants.^86^ Similarly, biochar-based organophosphate fertilizers can promote phosphorus availability in soil.^87^ These applications offer potential benefits in nutrient management and soil conditioning, but their long-term environmental impacts still needs to be better understood, for example, in relation to soil accumulation.

## 3 Applications of Carbonaceous Materials Derived from Biomass

Biomass-derived carbons have played an increasingly important role in the development of sustainable solutions, with applications spanning several key areas in both academic research and industry. Some of the main applications of these materials are illustrated in Figure 6.

**Figure 6:**
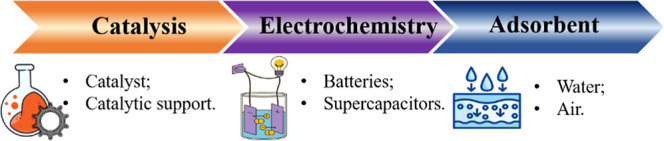
Representative applications of biomass-derived char.

### 3.1 Catalysis

Biomass and biomass-derived residues have emerged as excellent precursors for the production of heterogeneous catalysts, serving both as catalyst supports and as precursors for active phases and as supports for metal species. In recent years, the development of biomass-derived catalysts has increased significantly, driven by their low cost and reduced environmental impact, which are consistent with the principles of green chemistry and the circular economy.^88^ Furthermore, these catalysts support a wide range of industrial processes, from the synthesis of fine chemicals to large-scale commodity production, thereby promoting innovation, sustainability, and efficiency.^34,89^

The production of carbonaceous materials from biomass generally begins with a carbonization process, with pyrolysis and hydrothermal carbonization (HTC) being the most commonly employed methods, producing biochar and hydrochar, respectively.^9,90^ Figure 7 summarizes these two processes.

**Figure 7:**
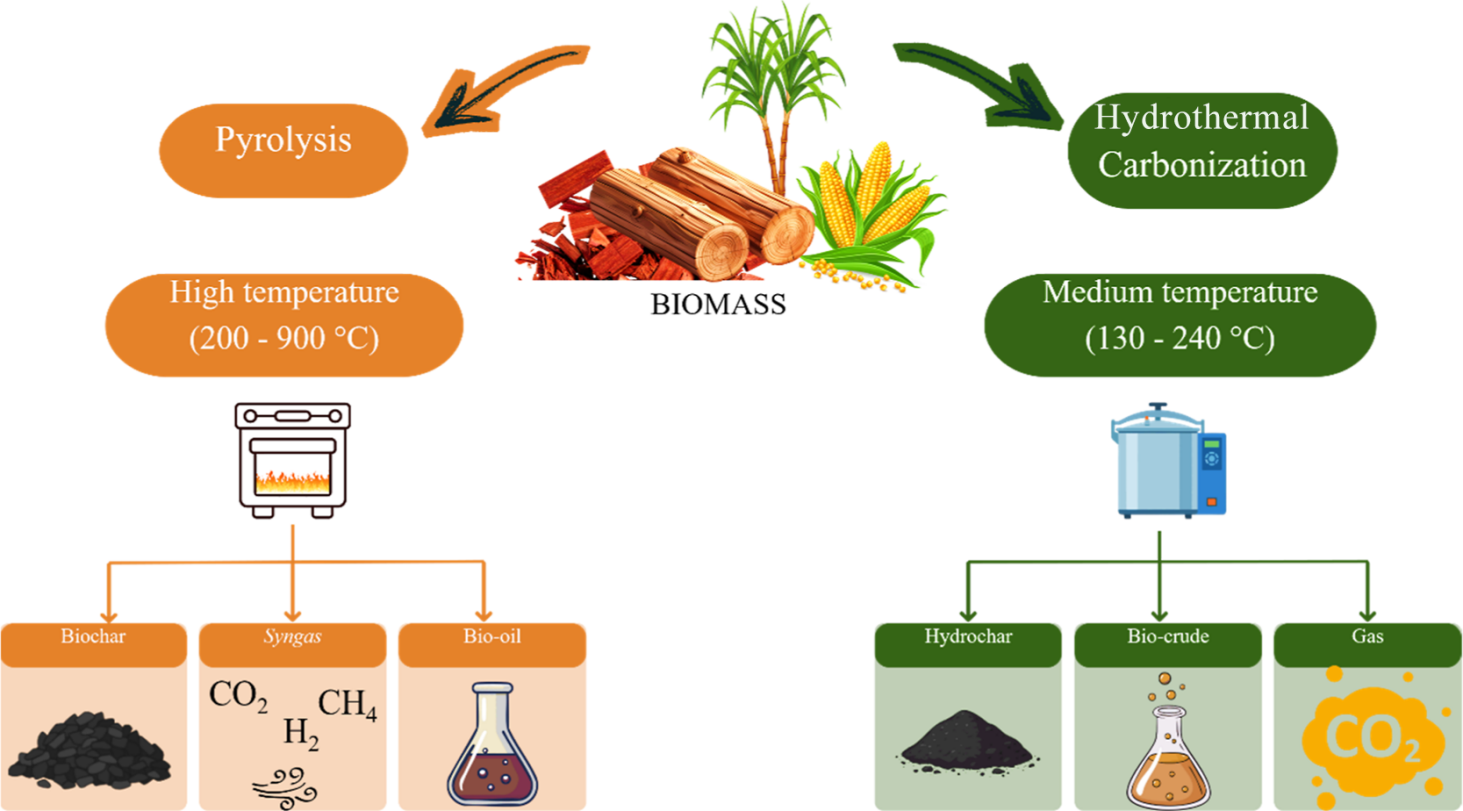
Conversion pathways from biomass to bio/hydrochar.

Pyrolysis is typically carried out at higher temperatures (200–900 °C) under an atmosphere containing little or no oxygen,^9,91^ whereas HTC is performed in an aqueous medium at temperatures up to approximately 240 °C, eliminating the need for prior drying of the precursor.^11,92–94^ During these processes, reactions such as dehydration, condensation, polymerization, and aromatization promote the formation of amorphous and porous carbonaceous structures. In addition, increasing pyrolysis temperature intensifies aromatization and graphitization reactions, leading to the development of graphite-like domains and defective carbon structures, whereas HTC-derived materials generally exhibit predominantly amorphous carbon characteristics.^95^

HTC-derived carbons generally exhibit low porosity, therefore, chemical activation steps using H_3_PO_4_ or KOH are often employed to increase the surface area and promote a more uniform pore size distribution.^90,96^ Moreover, the textural properties of hydrochars are strongly dependent on carbonization conditions, since prolonged reaction times and higher temperatures may induce structure densification and pore collapse, reducing surface area and pore volume.^95^ One-pot sulfonation, or sulfonation performed after carbonization, introduces –SO_3_H groups covalently bonded to the carbonaceous matrix, imparting strong Brønsted acidity and high catalytic efficiency.^10,97,98^

The acidity of biomass-derived sulfonated carbons primarily arises from the presence of –SO_3_H, –COOH, and phenolic –OH groups,^99^ exhibiting acidity comparable to that of sulfonic acid resins such as Amberlyst 15.^100^ In addition, these solid acid catalysts have the potential to replace conventional homogeneous acid catalysts, such as H_2_SO_4_, HCl, HNO_3_ and H_3_PO_4_, which are associated with drawbacks including corrosiveness, difficult separation from the reaction medium, and the need for post-treatment of acidic waste streams.

The applicability of these heterogeneous catalysts has been widely demonstrated in esterification, transesterification, and acetalization reactions. Sulfonated carbons derived from agricultural residues have shown high yields in the production of biodiesel and oxygenated derivatives, along with good stability and reusability.^11,101–103^ These catalytic platforms are particularly relevant for the upgrading of biomass-derived platform molecules such as furfural and HMF due to their tunable acidity, porous structure, and potential for sustainable catalytic applications.

In addition to carbonaceous materials, mineral-rich residues such as shells, eggshells, ashes, and sludges have been employed as precursors for metal oxides, particularly CaO, MgO, and other mixed oxides. Biomass can also serve as a source of mixed oxides, being used either directly as a catalyst or as support/precursor for metal impregnation.^104,105^ These materials are predominantly synthesized via calcination (300–1000 °C), following washing, drying, and grinding steps.^106,107^ Due to their high CaCO_3_ content, eggshells and other calcareous residues represent promising sources of catalytic CaO, which has been successfully applied in biodiesel production from waste oils.^106,108,109^

Biomass can also act as a support for metal impregnation, enhancing structural stability and reducing the leaching of active species. Composites derived from coal ash or sugar cane bagasse impregnated with metal oxides have demonstrated high performance and satisfactory reusability in transesterification reactions.^110,111^

Overall, the catalytic utilization of biomass integrates waste valorization, cost reduction, and mitigation of environmental impacts, thereby contributing strategically to the development of more sustainable chemical processes.

### 3.2 Supports for Catalysts

Carbonaceous materials derived from biomass stand out due to their versatility and, when used as supports, are capable of immobilizing catalytically active species, such as metals. This enables the transformation of homogeneous systems into heterogeneous ones, simplifying separation, recovery, and reuse steps, while also enhancing stability and selectivity in a variety of chemical processes.^112^

Carbons obtained from renewable resources have emerged as efficient supports, promoting the immobilization of various catalytic species, as well as improved reaction rates, selectivity, stability, and functionality across diverse reaction systems. Table 1 summarizes applications employing carbonaceous matrices from different sources as supports for metals in important chemical processes.

**Table 1:** Biochar as a Catalyst Support in Sustainable Catalytic Processes^c^

char feedstock	active supported material	surface area catalyst	applications	performance	ref
neem leaves	Cu-nanoparticles/MFe_2_O_4_	11.2–29.3 m^2^ g^–1^	formation of carbon-nitrogen bonds	40–85% conversion	^113^
pruning waste	CuO	-	degradation of 4-nitrophenol	100% degradation	^114^
olive stone	Cu/Ni	-	degradation of methyl orange	75% yield	^115^
straw	FeCo-MOF	91.821 m^2^ g^–1^	degradation of tetracycline	97.7% efficiency	^116^
oak sawdust	Bi_2_WO_6_ ^b^	27.91 m^2^ g^–1^	photodegradation of chlorpyrifos	99.9% efficiency	^117^
rice husk	Fe/Ni	295.14 m^2^ g^–1^	toluene removal	92.76% efficiency	^118^
pine sawdust	Ni/Fe	330 m^2^ g^–1^	syngas production and tar removal	increased synthesis gas yield by > 82%	^119^
wheat straw	Ni^a^	87 m^2^ g^–1^	CO_2_ methanation	up to approximately 65% conversion and selectivity	^120^
vineyard pruning residue	Co^b^	116.1 m^2^ g^–1^	hydrogenation furfural	80% conversion; 70% selectivity	^121^
olive stone	Ni	1073.1 m^2^ g^–1^	CO_2_ methanation	72.5% conversion; 95.46% selectivity	^122^
Cumaru sawdust	CaO	777.7 m^2^ g^–1^	biodiesel synthesis	98% yield	^123^
tanin	Co/Ni	541 m^2^ g^–1^	hidrogen evolution	hydrogen generation rate 6091.51 mL min^–1^ g^–1^	^13^
rice husk	Pd	2635 m^2^ g-^1^	benzaldehyde hydrogenation	100% conversion; 81% yield	^124^
coconut shell SiO_2_	Cu/Zn	1376–1420 m^2^ g^–1^, 250 m^2^ g^–1^	CO_2_ hydrogenation	<10%, <5%	^125^

a Material supported on CeO_2_-doped activated biochar.

b Materials supported on nitrogen-doped biochar.

c Yield.

Magnetic composites (Cu@KF-C/MFe_2_O_4_) based on activated carbon modified with metal nanoparticles (M = Cu, Co, Cu, Ni and Zn) have attracted considerable attention due to their catalytic efficiency in C–N coupling reactions, ease of magnetic separation, excellent stability, and reusability. The performance of these systems depends on the dispersion of the metallic phase and the physicochemical properties of the carbon support. For example, in a series of Cu-based magnetic composites, variations in BET surface area (11.2–35.4 m^2^ g^–1^) and pore volume was directly correlated with catalytic performance, with the CoFe_2_O_4_-containing material exhibiting superior activity due to its more developed porous structure.^113^ This suggests that, even in systems with relatively low surface areas, differences in pore architecture and accessibility can significantly influence catalytic efficiency.

In the environmental field, metals supported on carbonaceous matrices have been successfully applied in the degradation of aqueous pollutants, such as 4-nitrophenol, 4-aminophenol^114^ and methyl orange,^115^ as well as in the removal of tetracycline (>97%).^116^ While these systems generally exhibit high efficiencies, differences in reaction conditions (e.g., pH, oxidant presence, catalyst loading, time and temperature) make direct comparison of catalytic performances difficult. Additionally, these systems exhibit high efficiency in the removal of volatile organic compounds, such as toluene (>92%)^118^ and tar,^118,119^ reinforcing their potential for environmental remediation. Nevertheless, catalyst deactivation due to surface fouling and pore blockage remains an important limitation that is often not systematically addressed.

Among studies investigating the incorporation of metals into carbonaceous matrices, those employing doped biochar have demonstrated superior performance. For instance, carbonaceous matrices doped with cerium oxide^120^ and nitrogen,^121^ showed enhanced performance compared to their nondoped counterparts. This improvement is generally attributed to changes in electronic structure, surface functionality, and metal dispersion. However, the extent of these effects depends on the nature and level of doping.

In the energy context, metals supported on various biochars exhibit remarkable performance in the production of high-value fuels, such as CH_4_, biodiesel, and H_2_. Biochar derived from wheat straw and olive pits has been used as a support for metals, showing efficiency in CH_4_ production from CO_2_.^120,122^ Mesoporous carbons derived from tannins have been used to support Co/Ni nanoparticles for H_2_ generation from NaBH_4_, exhibiting high efficiency and excellent performance over six reuse cycles.^13^ Furthermore, bimetallic catalysts supported on biochar show high performance in syngas production with a high hydrogen fraction.^119^ As observed in Table 1, the wide range of surface areas and catalytic performance highlights that activity is governed not solely by porosity but also by pore structure, surface functionality, and support-metal interactions, which are strongly influenced by precursor selection and processing conditions.

Beyond the chemical transformations discussed above, metal-doped carbonaceous catalysts have also demonstrated outstanding performance in hydrogenation processes and electrocatalytic applications.^117,120,121^ However, long-term stability, metal leaching, and regeneration efficiency remain critical issues that require investigation, particularly under industrially relevant conditions.

From this perspective, the valorization of plant, agricultural, and animal-derived residues as catalytic precursors contributes to cost reduction and mitigation of environmental impacts, aligning with the principles of the circular economy. Nevertheless, challenges related to feedstock variability, reproducibility, and scale-up must be addressed to fully realize their potential. Thus, the use of biomass as a catalyst support integrates technical performance, waste valorization, and economic feasibility, representing a sustainable approach for the development of low-impact chemical processes.

### 3.3 Electrochemistry

Functionalized carbonaceous materials represent a key pathway in the development of sustainable electrochemical systems, combining high performance with the principles of the circular economy. These materials can exhibit high surface area, tunable porosity, good hydrophilicity, heteroatom incorporation, and high electrical conductivity, which are essential properties for applications in batteries and supercapacitors. In addition, are particularly attractive due to their abundance, renewable origin, low cost, and morphological versatility, often displaying physicochemical properties comparable to or even superior to those of commercial carbons and conventional metals.^126–128^

A wide range of biomass sources have emerged as effective precursors for the production of carbons intended for electrochemical systems, including cotton,^129^ peanut shells,^130^ olive leaves,^14^ bamboo and algae,^131^ corn cobs,^128^ and kombucha.^132^ These materials exhibit suitable structural compositions for the formation of porous carbons with properties favorable for charge storage. Table 2 summarizes the main characteristics of these biomass sources and the correlation between their properties and electrochemical performance after carbonization via pyrolysis.

**Table 2:** Characteristics of Different Biomasses Used as Carbonaceous Precursors in Electrochemical Systems

char feedstock	surface area (m^2^ g^–1^)	activation	application	specific capacitance /specific capacity	current density (A g^–1^)	ref
cotton	-	-	batteries	272 mAh g^–1^	0.05	^129^
peanut shell	1401.45	FeCl_3_/MgCl_2_ and CO_2_	supercapacitors	247.28 F g^–1^	1.0	^130^
olive leaves	839.33	-	batteries/supercapacitors	331.0 and 265.37 mAh g^–1^ /169.6 F g^–1^	0.5	^14^
bamboo and seaweed	1285.15	CH_3_COOK	supercapacitors	320.4 F g^–1^	0.5	^131^
corn cob	1612.86	KMnO_4_	supercapacitors	691.05 F g^–1^	0.5	^128^
kombucha	917.00	KOH	supercapacitors	326 F g^–1^	1.0	^132^

Several studies have demonstrated the potential of these precursors for applications in energy storage devices. Cotton textile waste has been converted into hard carbon for sodium-ion battery anodes, delivering a specific capacity of 272 mAh g^–1^ at 50 mA g^–1^ and retaining 98% of its capacity after 100 cycles.^129^ Similarly, activated carbons derived from peanut shells exhibited high surface area and a specific capacitance of 247 F g^–1^, along with good cycling stability in supercapacitors. The activation with ZnCl_2_ and FeCl_3_ favors the formation of micropores, whereas the presence of MgCl_2_ promotes the generation of mesopores.^130^ Carbons derived from olive leaves have also demonstrated excellent performance across different devices, exhibiting capacities of 331 mAh g^–1^ in lithium-ion batteries and 265 mAh g^–1^ in sodium-ion batteries, as well as a capacitance of 169.6 F g^–1^ in supercapacitors.^14^

Other studies have explored activation and doping strategies to improve the electrochemical performance of these materials. The combination of bamboo with spirulina enabled the production of porous carbons doped with N, O, and S, achieving a specific capacitance of 320.4 F g^–1^ and high cycling stability.^131^ Carbon materials derived from corn cobs and activated with KMnO_4_ exhibited a surface area of 1612.89 m^2^ g^–1^ and a capacitance of 691.05 F g^–1^, demonstrating excellent performance in supercapacitors.^128^ Likewise, hierarchical carbons obtained from kombucha activated with KOH displayed a capacitance of 326 F g^–1^ and excellent cycling stability, highlighting the potential of this biomass as a precursor for energy storage devices.^132^

In general, the electrochemical performance of the aforementioned materials is strongly related to surface area and pore size distribution. Direct carbonization of biomass, although capable of producing carbonaceous materials, often does not generate sufficiently developed porous structures for electrochemical applications. Therefore, control of the microstructure, including pore shape, size, and connectivity, is essential for optimizing ion adsorption and charge transport.^127,128^ Accordingly, activation processes are widely employed to enhance surface area and develop hierarchical porous networks. Activation can be classified as chemical, physical, or physicochemical.^127,128^

Chemical activation is the most widely used method due to its high efficiency in generating highly porous structures. In this process, activating agents such as KOH, metal chlorides (FeCl_3_/ZnCl_2_, FeCl_3_/MgCl_2_, and ZnCl_2_/MgCl_2_), CH_3_COOK, KMnO_4_, K_2_FeO_4_, (NH_4_)_2_HPO_4_, and K_2_CO_3_ are impregnated into the precursor and subjected to carbonization under an inert atmosphere.^128,130–132^ During heating, dehydration, partial oxidation, and internal etching reactions occur within the carbon matrix, resulting in pore formation and expansion. After thermal treatment, the materials are washed to remove residual activating agents and neutralize the pH.^127,128^

Physical activation is based on the use of oxidizing gases such as carbon dioxide, steam, air, or gas mixtures at high temperatures to promote controlled oxidation of the carbon matrix, thereby opening and widening existing pores. Although considered an environmentally friendly approach due to the absence of chemical washing steps, this method generally requires higher temperatures (350–1000 °C) and yields lower surface areas compared to chemical activation.^127,128^

Physicochemical activation combines both strategies, typically involving an initial chemical activation step followed by treatment with oxidizing gases. This strategy enables finer control over the porous architecture, favoring the production of hierarchical carbons with superior properties for electrochemical applications, although it involves greater operational complexity.^128^

In summary, the activation process is crucial not only for increasing surface area but also for generating an interconnected pore network that governs ion diffusion and charge storage. The balance between micropores (ion storage) and mesopores (rapid ion transport) is essential to maximize capacitance and cycling stability in supercapacitors and batteries.^127,130^

Building on these considerations, the structural features that govern electrochemical performance, such as high surface area, hierarchical porosity, and surface functionalization, also underpin the effectiveness of biomass-derived carbons in environmental applications. In particular, the balance between micropores and mesopores, which is critical for ion storage and transport, also influences adsorption capacity and pollutants mass transfer. Thus, the same characteristics that enhance charge storage and catalytic behavior can be extended to the adsorption and removal of contaminants from water and air, highlighting the versatility of these materials across energy and environmental domains.

### 3.4 Adsorbents

Biomass-derived materials have also emerged as promising adsorbents, particularly for the removal of environmental pollutants such as heavy metals and dyes from wastewater, which are often difficult to remove using conventional treatment methods.^133^ The removal of these contaminants is essential for environmental protection and human health.^134,135^

Various methods have been employed for the removal of organic pollutants and dyes, including coagulation, biological treatment, membrane separation, advanced oxidation processes, and adsorption.^133,134,138^ However, these approaches often present significant limitations, such as high operational costs, long treatment times, and the generation of secondary waste.^133,135^ In this context, adsorption stands out as an efficient alternative due to its simplicity, low operational cost, and high removal efficiency, as well as its possibility of adsorbent regeneration.^134,135,138^

Despite the wide range of commercially available adsorbents, their regeneration process can be costly.^133^ Accordingly, biomass-derived adsorbents have attracted increasing attention due to their renewability, low cost, and tunable surface properties.^133,135,138^ Chemical modification of these materials with metals or nanomaterials can improve properties such as surface area, chemical functionality, active sites, and pore size distribution, thereby improving their adsorption performance.^138^

Among these materials, carbonaceous adsorbents stand out in wastewater purification due to their high porosity and the presence of surface functional groups, such as phenolic, carboxyl, and carbonyl groups, which can interact with pollutants through chemical and physical adsorption mechanisms.^136^ Activated carbon, in particular, is widely recognized for its high surface area, well-developed porous structure, and good reusability.^135,136^

As summarized in Table 3, biomass-derived adsorbents exhibits a wide range of surface area and adsorption capacities, reflecting the strong influence of precursor type and activation method on material properties. For instance, ultramicroporous activated carbons derived from lignocellulosic residues show high CO_2_ adsorption capacities, which are closely related to their pore size distribution rather than surface area alone.^138,137,139^ In contrast, metal-modified adsorbents have demonstrated enhanced performance in dye removal, highlighting the role of surface functionalization and specific interactions between the adsorbent and the pollutant.^134,140^

**Table 3:** Biomass-Derived Adsorbents for Contaminants and Gas Removal

char feedstock	material adsorbent	surface area	application	performance	relevant observations	ref
green pods Cassia fistula	metallic nanoparticles (Fe, Ni, Cu)	18.33–62.36 m^2^ g^–1^	malachite green	662 mg g^–1^ (Fe); 458 mg g^–1^ (Ni); 1355 mg g^–1^ (Cu); 456 mg g^–1^ (no metal)	up to 20× greater than commercial adsorbents	^134^
Coccinia grandis	bionanocomposite modified with Cu	15.2 m^2^ g^–1^	malachite green	99% removal; 49 mg g^–1^	superior to commercial activated carbon	^140^
rice rusk	N-doped mesoporous AC	1226 m^2^ g^–1^	CO_2_ adsorption	5.43 mmol g^–1^	stable regeneration after 12 cycles	^139^
slash pine (wood)	ultramicroporous activated carbon	906–1384 m^2^ g^–1^	CO_2_ adsorption	4.22–5.44 mmol m^–2^	high microporosity and performance	^138^
date sheets	ultramicroporous activated carbon	2112–3337 m^2^ g^–1^	CO_2_ adsorption	up to 22 mmol g^–1^	surface specific area up to 3337 m^2^ g^–1^; adsorption dependent on pore size/area; reusability over 4 cycles	^137^
^a^	Si-modified AC (Si/AC)	AC: 1698 m^2^ g^–1^ Si/AC–0.1: 1495 m^2^ g^–1^	toluene	AC: 99 mg g^–1^; Si/AC–0.1: 130 mg g^–1^; ∼80% efficiency after 5 cycles	greater microporosity; high temperature stability	^141^

a Commercial activated carbon (AC).

However, direct comparison of adsorption performance across studies remains challenging due to variations in experimental conditions (e.g., pollutant concentration, pH, temperature, and contact time). Although high adsorption capacities are frequently reported, key aspects such as regeneration efficiency, long-term stability, and scalability are not systematically evaluated, with most studies reporting only a limited number of reuse cycles (Table 3). In addition, the surface area of biomass-derived adsorbents varies widely, often within or exceeding the typical range for activated carbons (500–1500 m^2^ g^–1^).^142^ However, higher surface area does not necessarily translate into superior adsorption performance, as factors such as pore size distribution and surface functionality play a decisive role.

Therefore, while biomass-derived adsorbents represent a sustainable and versatile class of materials, further efforts are needed to establish standardized evaluation protocols and to better understand the relationship between structure, surface chemistry, and adsorption performance. In this context, comparative studies between commercial and biomass-derived adsorbents under standardized conditions are particularly important to assess their practical potential.

## 4 Challenges and Future Perspectives

The selective conversion of biomass, particularly lignocellulosic biomass, into platform molecules such as furfural and HMF is often limited by side reactions, including polymerization and humin formation,^25^ which reduce yields and complicate downstream purification. The development of catalysts capable of controlling these competing pathways remains a significant challenge.

Although biomass-derived sulfonated carbon catalysts have demonstrated promising catalytic activity, their long-term stability, resistance to functional group leaching, and efficient reusability remain major limitations. For instance, in reactions conducted at elevated temperatures, sulfonic groups tend to detach from the carbon framework at temperatures above 170 °C, as evidenced by thermogravimetric analysis.^143^ Therefore, strategies aimed at improving the anchoring of acidic groups and controlling surface chemistry are essential.

Another important aspect concerns the structural control of biomass-derived carbons. The relationship between porosity, surface functional groups, and catalytic performance is not yet fully understood. From a technological perspective, the scalability of hydrothermal carbonization and pyrolysis processes must also be addressed, as variations in biomass composition (cellulose, hemicellulose, and lignin) can significantly affect material properties.^11^

In light of these considerations, there remains substantial opportunities remain for research focused on integrating biomass conversion processes within biorefineries and developing advanced carbon materials with tunable surface chemistry and enhanced catalytic stability.

## 5 Conclusions

In summary, this review advances the understanding of lignocellulosic biomass valorization by systematically connecting the production of platform molecules, such as furfural and HMF, with the development of biomass-derived carbonaceous materials. Considering these fields within a unified framework enables the identification of converging design principles and relationships that are not readily apparent when addressed separately. Despite substantial progress in catalytic conversion and material synthesis, key limitations persist. Variability in experimental conditions, the absence of standardized benchmarking protocols, and the limited evaluation of catalyst stability and recyclability hinder meaningful comparison across studies and obscure the identification of scalable systems. In addition, challenges related to catalyst deactivation, process integration, and product separation remain critical barriers to practical implementation.

The analysis presented here highlights underexplored opportunities at the interface of biomass-derived intermediates and carbon-based materials, particularly in the rational design of multifunctional systems and the elucidation of structure–performance relationships. Advancing the field will require the development of robust and regenerable catalysts, the adoption of consistent evaluation criteria, and the integration of catalytic and separation processes, supported by techno-economic analysis.
